# Effect of Body Mass Index on Breast Cancer during Premenopausal and Postmenopausal Periods: A Meta-Analysis

**DOI:** 10.1371/journal.pone.0051446

**Published:** 2012-12-07

**Authors:** Zahra Cheraghi, Jalal Poorolajal, Tahereh Hashem, Nader Esmailnasab, Amin Doosti Irani

**Affiliations:** 1 Department of Epidemiology & Biostatistics, School of Public Health, Hamadan University of Medical Sciences, Hamadan, Iran; 2 Research Center for Health Sciences and Department of Epidemiology & Biostatistics, School of Public Health, Hamadan University of Medical Sciences, Hamadan, Iran; 3 Fatemieh Hospital, Hamadan University of Medical Sciences, Hamadan, Iran; 4 Department of Community Medicine, School of Medicine, Kurdistan University of Medical Sciences, Kurdistan, Iran; University of Cordoba, Spain

## Abstract

**Objective:**

There is no universal consensus on the relationship between body mass index (BMI) and breast cancer. This meta-analysis was conducted to estimate the overall effect of overweight and obesity on breast cancer risk during pre- and post-menopausal period.

**Data Sources:**

All major electronic databases were searched until April 2012 including Web of Knowledge, Medline, Scopus, and ScienceDirect. Furthermore, the reference lists and related scientific conference databases were searched.

**Review Methods:**

All prospective cohort and case-control studies investigating the association between BMI and breast cancer were retrieved irrespective of publication date and language. Women were assessed irrespective of age, race and marital status. The exposure of interest was BMI. The primary outcome of interest was all kinds of breast cancers confirmed pathologically. Study quality was assessed using the checklist of STROBE. Study selection and data extraction were performed by two authors separately. The effect measure of choice was risk ratio (RR_i_) and rate ratio (RR_a_) for cohort studies and odds ratio (OR) in case-control studies.

**Results:**

Of 9163 retrieved studies, 50 studies were included in meta-analysis including 15 cohort studies involving 2,104,203 subjects and 3,414,806 person-years and 35 case-control studies involving 71,216 subjects. There was an inverse but non-significant correlation between BMI and breast cancer risk during premenopausal period: OR = 0.93 (95% CI 0.86, 1.02); RR_i_ = 0.97 (95% CI 0.82, 1.16); and RR_a_ = 0.99 (95% CI 0.94, 1.05), but a direct and significant correlation during postmenopausal period: OR = 1.15 (95% CI 1.07, 1.24); RR_i_ = 1.16 (95% CI 1.08, 1.25); and RR_a_ = 0.98 (95% CI 0.88, 1.09).

**Conclusion:**

The results of this meta-analysis showed that body mass index has no significant effect on the incidence of breast cancer during premenopausal period. On the other hand, overweight and obesity may have a minimal effect on breast cancer, although significant, but really small and not clinically so important.

## Introduction

Breast cancer is the most common cancer in women both in the developed and the developing countries, comprising 16% of all female cancers. It is estimated that breast cancer led to 519,000 death in women in 2004 [1]. Although breast cancer is thought to be a common cancer in the developed countries, a majority (69%) of all breast cancer deaths occurs in developing world. Indeed, increase life expectancy, increase urbanization and adoption of western lifestyles have increased the incidence of breast cancer in the developing countries [1], [2]. A recent study indicated that breast cancer is the leading cause of cancer and cancer related mortality in woman worldwide so that cause-specific mortality rate increases with age among postmenopausal women with hormone receptor-positive breast cancer [3].

The etiology of breast cancer is not well known. However, several risk factors have been suggested to have an influence on the development of this malignant tumor including genetic, hormonal, environmental, sociobiological and physiological factors [2]. Weight gain and obesity is another potential risk factor which may influence the incidence of breast cancer. There are numerous observational studies which have investigated the correlation between obesity and breast cancer. However the results are inconsistent. Some researchers believe that body mass index greater than 30 may increase the risk of breast cancer both in pre- and postmenopausal periods [4]–[7] whereas others claim that obesity may reduce the risk of breast cancer during premenopausal period but increase the risk during postmenopausal period [8]–[11].

There is no universal consensus on the relationship between BMI and breast cancer. To date, a few meta-analyses have been conducted to estimate a summary measure of the effect size of overweight and obesity on breast cancer. However, these studies were limited to the English language studies cited by Medline [12], [13]. Thus, the present up-to-date meta-analysis was conducted to assess the results of both cohort and case-control studies addressing the correlation between BMI and breast cancer cited by all major international electronic databases in order to estimate the overall effect of body mass index (BMI) on breast cancer risk.

## Materials and Methods

### Searching

We planned to include cohort and case-control studies addressing the association between body mass index and breast cancer. We developed a search strategy using and combing a set of keywords including breast cancer, body mass index, waist hip ratio, obesity, overweight, body size, cohort studies, case-control studies, and observational studies. We search all major electronic databases including Web of Knowledge (January 1945 to April 2012); Medline (January 1950 to April 2012); Scopus (January 1973 to April 2012); ScienceDirect (January 1823 to April 2012).

In order to find additional references, we scanned the reference lists of all retrieved studies. In addition, we contacted authors of retrieved studies for additional unpublished studies. Furthermore, the following conference databases were searched for unpublished data until April 2012:

- American Society of Clinical Oncology; available from: www.asco.org
- American Cancer Society; available from: www.cancer.org
- International Agensy for Research on Cancer; available from: www.iarc.fr

### Criteria for including studies

We included prospective cohort studies and case-control studies investigating the association between BMI and breast cancer irrespective of publication date and language. The retrospective cohort and matched case-control studies were excluded. We included those apparently healthy women irrespective of age, race and marital status. The exposure of interest was obesity and overweight using BMI. The term ‘BMI’ is a commonly used index to classify overweight and obesity in adults and is defined as the weight in kilograms divided by the square of the height in meters (kg/m^2^). Based on the World Health Organization classification [14] BMI<18.5 is considered as underweight, 18.5≤BMI<25 as normal weight, 25≤BMI<30 as overweight, and BMI≥30 as obese. The primary outcome of interest was breast cancer of any type which was confirmed pathologically. We planned to include all kinds of breast cancers irrespective of pathological characteristics and stage of the tumor.

### Data collection and validity assessment

Two authors (ZC and ADI) read the retrieved publications separately in order to identify the studies that would meet the inclusion criteria of this review. The authors were not blinded to the authors' names of the publications, journals, and results. Any disagreements were resolved by adjudication with a third author (JP). The inter-authors reliability based on kappa statistics was 85%.

Two authors (ZC and ADI) extracted the data from the included studies. The variables which were extracted for data analysis included study design, year and location of study conduction, sample size, number of outcomes, mean age, gender, and body mass index. The extracted data were entered in the electronic data sheet. In cases of missing data or need for clarification, study authors were contacted.

We intended to assess the risk of bias of the included studies using the recommended checklist of STROBE [15] Two authors (ZC and ADI) assessed the studies independently. The items which were evaluated for judgment about cohort studies included (a) state specific objectives of the study; (b) present key elements of study design; (c) give the eligibility criteria; (d) clearly define exposure (here obesity and overweight); (e) clearly define outcome (here breast cancer); and (f) explain how loss to follow-up was addressed. The last item was merely evaluated for cohort studies.

### Measures of exposure effect and data analysis

The effect measure of choice for cohort studies was risk ratio (RR_i_) and rate ratio (RR_a_) and that of case-control studies was odds ratio (OR). RR_i_ was defined as the probability of a disease in exposed people to the probability of the disease in unexposed people in a cohort study. RR_a_ was defined as the proportion of a disease in exposed people to a specified person-year (a statistical measure representing one person at risk of development of a disease during a period of 1 year) in a cohort study. OR was the proportion of the exposed population in whom disease has developed over the proportion of the unexposed population in whom disease has developed in a case-control study [16].

Meta-analysis was performed to obtain summary measure with 95% confidence interval (CI). Both Review Manager 5 [17] and Stata 11 (StataCorp, College Station, TX, USA) were employed for data analysis. Data were analyzed and the results were reported using random effect models [18].

### Heterogeneity and publication bias

We explored statistical heterogeneity using the chi-squared (χ^2^ or Chi^2^) at the 5% significance level (*P*<0.05). We quantified inconsistency across studies results using I^2^ statistic [19]. We also estimated the between-study variance using tau-squared (τ^2^ or Tau^2^) statistic [20]. We used funnel plot to investigate publication bias visually [20] as well as Begg's [21] and Egger's [22] tests to assess publication bias statistically.

## Results

### Description of studies

We retrieved 9163 studies up to April 2012, including 8370 references through searching electronic databases, 241 references through conference databases, 546 references through checking reference lists, and six references through personal contact with studies' authors. Of 9163 retrieved references, 2680 references were excluded because of duplication, 6273 references did not relate to the objective of this review, and 160 references did not meet the eligibility criteria. Eventually, we included 50 studies in the meta-analysis including 15 cohort studies [4]–[6], [10], [15], [23]–[32] involving 2,104,203 people and 3,414,806 person-years and 35 case-control studies [8], [9], [33]–[65] involving 71,216 people. Some case-control and cohort studies evaluated breast cancer during premenopausal period, some during postmenopausal period and some during both periods. Thus, some studies presented only once and some others presented more than once in forest plots. However, the total number of 35 case-control and 15 cohort studies were included in meta-analysis.

### Effect of exposure

**Figure 1:**
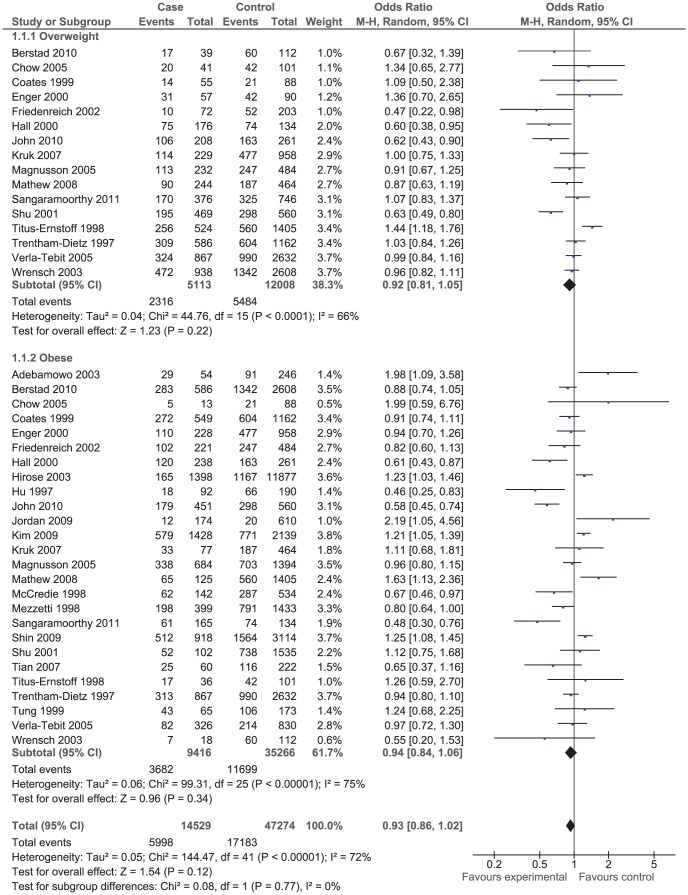
Forest plot of odds ratio estimates of breast cancer in premenopausal period by overweight and obesity.

**Figure 2:**
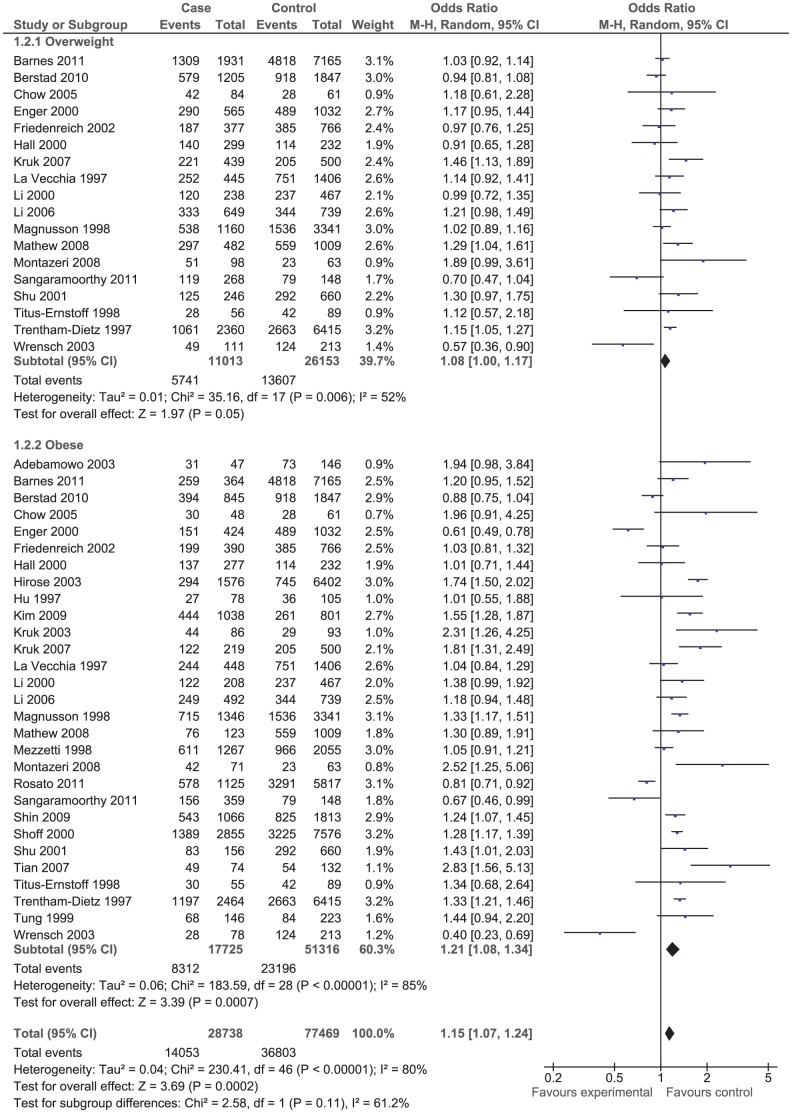
Forest plot of odds ratio estimates of breast cancer in postmenopausal period by overweight and obesity.

**Figure 3:**
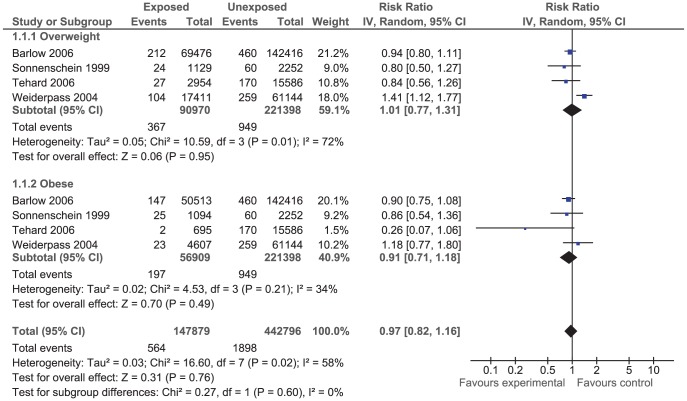
Forest plot of risk ratio estimates of breast cancer in premenopausal period by overweight and obesity.

**Figure 4:**
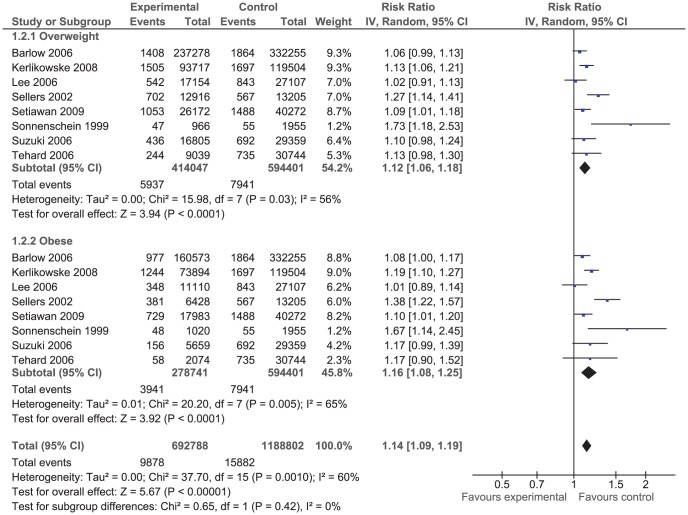
Forest plot of risk ratio estimates of breast cancer in postmenopausal period by overweight and obesity.

The effect of BMI on breast cancer risk during pre- and postmenopausal period was assessed using odds ratio (OR) (Figure 1 and 2) in case-control studies and using risk ratio (RR_i_) (Figure 3 and 4) and rate ratio (RR_a_) (not shown) in cohort studies.

The results of both case-control and cohort studies showed that increase in BMI during premenopausal period reduced the risk of breast cancer: OR = 0.93 (95% CI 0.86, 1.02); RR_i_ = 0.97 (95% CI 0.82, 1.16); and RR_a_ = 0.99 (95% CI 0.94, 1.05). That means women who were overweight or obese during premenopausal ages were at lower risk of breast cancer compared to women with normal weight although the observed inverse correlation was not statistically significant.

The results of both case-control studies and cohort studies showed that overweight and obesity in postmenopausal period increased slightly the risk of breast cancer: OR = 1.15 (95% CI 1.07, 1.24); RR_i_ = 1.16 (95% CI 1.08, 1.25); and RR_a_ = 0.98 (95% CI 0.88, 1.09). That means the women who were overweight or obese during postmenopausal period were significantly at higher risk of breast cancer.

The effect of overweight and obesity on the breast cancer risk was evaluated separately. According to the RR_i_ and OR values, obese women had lower risk of breast cancer compared to overweight women during premenopausal period. However, the correlation was reversed during postmenopausal period so that obese women were at higher risk of breast cancer compare to overweight women although the difference was not statistically significant.

We classified the cohort studies based on the selected items of the recommended checklist of STROBE into high-quality (33%) [10], [23], [25], [27], [66], intermediate-quality (54%) [4]–[6], [24], [28], [30]–[32] and low-quality (13%) [26], [29]. Similarly, the case-control studies were also classified into high-quality (74%) and intermediate-quality (26%). There was no statistically significant difference between the results of studies with different quality in both pre- and postmenopausal periods (Table 1).

**Table 1:** Effect of body mass index on incidence of breast cancer by quality of the studies, menopausal period, and study design.

	High Quality		Moderate Quality		Low Quality	
Period	Effect Size	Q-test	Effect Size	Q-test	Effect Size	Q-test
Study design	(95% CI)	*P* value	(95% CI)	*P* value	(95% CI)	*P* value
**Premenopausal**						
Case-control (OR)	0.93 (0.84, 1.02)	0.001	0.98 (0.79, 1.21)	0.020	No data	-
Cohort (RR_i_)	No data	-	0.97 (0.82, 1.16)	0.020	No data	-
Cohort (RR_a_)	1.02 (0.92, 1.13)	0.360	0.98 (0.91, 1.05)	0.010	No data	-
**Postmenopausal**						
Case-control (OR)	1.18 (1.08, 1.29)	0.001	1.08 (0.96, 1.22)	0.001	No data	-
Cohort (RR_i_)	1.01 (0.93, 1.10)	0.010	1.11 (1.06, 1.16)	0.020	1.22 (1.13, 1.32)	0.040
Cohort (RR_a_)	0.99 (0.90, 1.08)	0.001	0.96 (0.60, 1.54)	0.001	No data	-

### Heterogeneity and publication bias

The between studies heterogeneity was assessed using the Chi^2^ test and the I^2^ statistics. The results of Chi^2^ test indicated that case-control studies were significantly heterogeneous (*P*<0.001). The I^2^ statistics for premenopausal period was 72% and for that of postmenopausal period was 80% (Figures 1 and 2). On the contrary, the results of cohort studies were homogenous (*P* = 0.220). The I^2^ statistics for premenopausal period was 32.8% and for postmenopausal period was 34.5% (Figures 3 and 4).

Of 42 case-control studies (Figure 1) assessed the effect of BMI on breast cancer risk during premenopausal period, 24 studies reported negative associations (9 out of which were statistically significant) and 18 studies reported positive associations (7 out of which were statistically significant). Of 47 case-control studies (Figure 2) investigated the effect of BMI on breast cancer during postmenopausal period, 11 studies reported negative associations (5 out of which were statistically significant) and 36 studies reported positive associations (14 out of which were statistically significant). Of eight cohort studies (Figure 3) assessed the effect of BMI on breast cancer during premenopausal period, six studies reported negative associations (one out of which were statistically significant) and two studies reported positive associations (one out of which were statistically significant). Of 16 cohort studies (Figure 4) investigated the effect of BMI on breast cancer during postmenopausal period, no study reported negative associations while all 16 studies reported positive associations (9 out of which were statistically significant).

**Figure 5:**
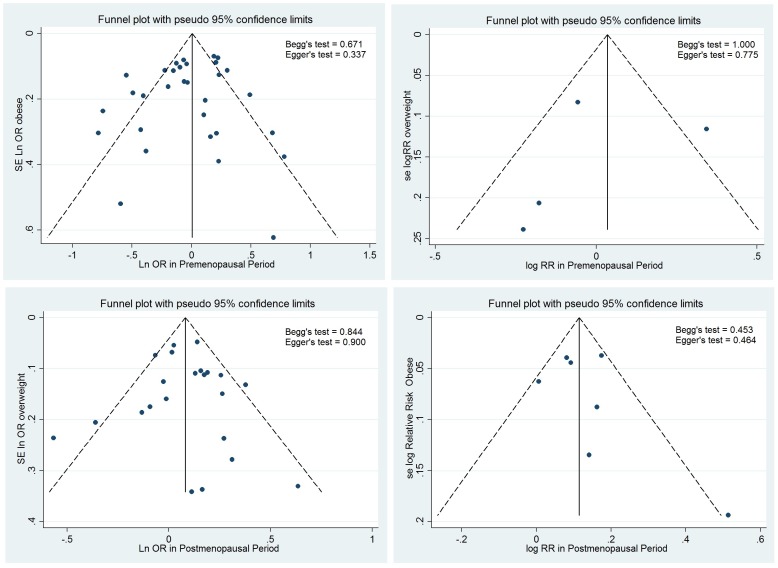
Funnel plot of included studies in pre postmenopausal and postmenopausal period by study design.

We assessed publication bias using the funnel plot as well as Begg's and Egger's tests. The studies scattered nearly symmetrically on both side of the vertical line reflecting absence of publication bias. The results of Begg's and Egger's tests for both OR and RR_i_ estimated in pre- and postmenopausal periods confirmed the absence of publication bias (Figure 5).

## Discussion

The results of this meta-analysis revealed that BMI during premenopausal period can decrease the risk of breast cancer by 0.07 although the association was not statistically significant. Contrary, increase in BMI during postmenopausal period can significantly increase the risk of breast cancer by 0.21. This evidence means that BMI is not a protective factor against breast cancer during premenopausal period. However, BMI is a weak but significant risk factor for breast cancer during postmenopausal period, although its effect is really small and not clinically important. The stronger the association, the more likely it is that the relation is causal while a weak association is more likely to be confounded although a weak association does not rule out causal connection [16]. Furthermore, increase in BMI during premenopausal period decreases the risk of breast cancer while increases the risk during postmenopausal period. This implies the presence of interaction between BMI and menopausal period. In such situation, the association should be assessed for each period separately and it is not reasonable to pool the data to estimate overall effect of BMI on breast cancer risk.

Suzuki et al [13] conducted a similar meta-analysis in order to assess the effect of BMI on breast cancer risk. They retrieved 31 references including nine cohort and 22 case-control studies indexed in Medline until December 2007. They reported that overweight during premenopausal period would decrease the risk of breast cancer; OR = 0.80 (95% CI 0.70, 0.92); while it might increase the risk of cancer during post menopausal period; OR = 1.89 (95% CI 1.52, 2.36). The results of their meta-analysis were rather different from ours. One reason was that we searched and retrieved the relevant references from all major international databases while Suzuki et al had searched only Medline database which might introduce selection bias in their results.

Another meta-analysis with same topic was conducted by Ryu et al [12]. They searched Medline database until 1999 and retrieved 12 case-control studies. They reported that overweight and obesity could increase the risk of breast cancer 1.56 times. However, they did not report the effect of body mass index on breast cancer during pre- and postmenopausal period separately. Furthermore, they had limited the search to the English language literatures indexed in Medline. This issue might also introduce selection bias in their results.

There was evidence of heterogeneity (small *P* value of Chi^2^ test and large I^2^ statistic) among the results of the included studies. However, care must be taken in the interpretation of the statistical tests for heterogeneity. The Chi^2^ test has low power when the sample size is small. On the other hand, the test has high power in detecting a small amount of heterogeneity that may be clinically unimportant when there are many studies in a meta-analysis [20]. Therefore, we can attribute major part of the observed heterogeneity in the results to the number of studies (including 15 cohort and 35 case-control studies) included in the meta-analysis and the large sample size (involving 2,104,203 participants in cohort studies and 71,216 participants in case-control studies).

Regardless of the effect of overweight and obesity on breast cancer, there are several well-documented risk factors for breast cancer. A familial history of breast cancer, reproductive factors associated with prolonged exposure to endogenous estrogens, such as early menarche, late menopause, late age at first childbirth are among the most important risk factors for breast cancer. Exogenous hormones such as oral contraceptive and hormone replacement therapy also exert a higher risk for breast cancer. Furthermore, alcohol use, and physical inactivity are among the modifiable risk factors for breast cancer [67].

There were a few limitations and potential biases in this meta-analysis. First, 15 studies seemed potentially eligible to be included in this meta-analysis but the full texts were not accessible. This issue may raise the possibility of selection bias. Second, we intended to assess the effect of other potential confounding variables such as onset of menarche, onset of menopause, smoking status, oral contraceptive consumption, and family history of breast cancer. However, these variables were not reported exactly in majority of the studies. Hence, we could not conduct subgroup analysis based on these variables. This issue may raise the possibility of the information bias. Despite its limitations, this meta-analysis could present strong evidence about the correlation between BMI and breast cancer by retrieving 9163 studies from all major databases and including 50 studies in the meta-analysis (15 cohort studies having 2,104,203 people and 3,414,806 person-years and 35 case-control studies involving 71,216 people).

In addition, our work brought some new information about the relationship between BMI and breast cancer, including (a) consolidation of the data to obtain summary measure of odds ratio, risk ratio, and rate ratio estimates regarding the effect of BMI on breast cancer; (b) non-significant inverse correlation between overweight and obesity and the incidence of breast cancer during premenopausal period; (c); significant direct correlation between overweight and obesity and the incidence of breast cancer during postmenopausal period; (d) the impact of various variables on the correlation between BMI and breast cancer such as studies designs, period of menopause, various types of BMI, and quality of the studies.

## Conclusion

The results of this meta-analysis showed that body mass index has no significant effect on the incidence of breast cancer during premenopausal period. On the other hand, overweight and obesity may have a minimal effect on breast cancer, although significant, but really small and not clinically so important.
